# Endoscopic ultrasound-assisted transmural cholecystoduodenostomy or cholecystogastrostomy as a bridge for per-oral cholecystoscopy therapy using double-flanged fully covered metal stent

**DOI:** 10.1186/s12876-016-0420-9

**Published:** 2016-01-19

**Authors:** Nan Ge, Siyu Sun, Shiwei Sun, Sheng Wang, Xiang Liu, Guoxin Wang

**Affiliations:** Endoscopy Center, Shengjing Hospital of China Medical University, No. 36, Sanhao Street, Shenyang, Liaoning Province 110004 China; Anesthesia Department, Shengjing Hospital of China Medical University, No. 36, Sanhao Street, Shenyang, Liaoning Province 110004 China

**Keywords:** Gallbladderstones, Endoscopic ultrasound, Metal stent

## Abstract

**Background:**

Laparoscopic cholecystectomy (LC) has become the ‘gold standard’ for the treatment of symptomatic gallstones. Innovative methods are being introduced, and these procedures include transgastric or transcolonic endoscopic cholecystectomy. However, before clinical implementation, instruments still need modification, and a more convenient treatment is still needed. Moreover, some gallbladders still have good functionality and cholecystectomy may be associated with various complications. The aim of this study was to evaluate the trans-gastrointestinal tract cholecystoscopy technique in the treatment of gallbladder disease without cholecystectomy.

**Method:**

Endoscopic ultrasound (EUS)-guided cholecystoduodenostomy or cholecystogastrostomy with the placement of a double-flanged fully covered metal stent was performed and endoscopic sphincterotomy (EST) was also performed during this procedure for those patients with accompanying common bile duct stones. One or two weeks later the stent was removed and an endoscope was advanced into the gallbladder via the fistula, and cholecystolithotomy or polyp resection was performed. Four weeks later gallbladder was assessed by abdominal ultrasound.

**Results:**

EUS guided cholecystoduodenostomy (*n* = 3) or cholecystogastrostomy (*n* = 4) with double flanged mental stent deployment was successfully performed in all of 7 patients. After the procedure, fistulas had formed in each of the patients and the stents were removed. Endoscopic cholecystolithotomy(7) and polyps resection(2) were successfully performed through the fistulas. Common bile duct stones were also successfully removed in 5 patients. The ultrasound examination of the gallbladder 4 weeks later showed no stones remaining and also showed satisfactory functioning of the gallbladder.

**Conclusion:**

The EUS-guided placement of a novel metal stent is a safe and simple approach for performing an endoscopic cholecystoduodenostomy or cholecystogastrostomy, which can subsequently allow procedures to be performed for treating biliary disease, including cholecystolithotomy.

## Background

Gallbladder stones and polyps are common benign diseases of the biliary tract system, often accompanied by common bile duct(CBD)stones [1, 2]. Laparoscopic cholecystectomy (LC) or LC plus laparoscopic common bile duct exploration (LCBDE) are the methods most widely used to treat symptomatic cases, since they are the most effective and minimally invasive techniques [3–5]. However, in some cases, the gallbladder still has good functionality and moreover cholecystectomy may be associated with various complications in some patients [6–8]. In previous animal studies we found that newly-developed lumen apposing metal stent could efficiently accomplish an anatomosis between the GI tract and the gallbladder [9]. The fistula formed by the stent may serve as an access point for endoscopic gallbladder disease treatment. The aim of this study was to evaluate this trans-gastrointestinal tract cholecystoscopy technique in treating gallbladder (GB) disease without the need for cholecystectomy. We also combined both endoscopic sphincterotomy (EST) and cholecystolithotomy into a one-session treatment for those patients who have both gallstones and common bile duct stones, which could potentially become another popular alternative to LC plus LCBDE [10].

## Method

### Patient

Retrospective chart review of patients treated with endoscopic cholecystoduodenostomy or cholecystogastrostomy in our hospital was performed. The inclusion criteria for the study were as follows: (1) symptomatic gallbladder stones (Present with upper abdominal pain, not relieved or exacerbated by movement, position, or bowel function. Atypical symptom such as chest pain, eructation, early satiety, dyspepsia. Or the symptom caused by complication of gallstone disease, including cholecystitis, cholangitis, or pancreatitis) were diagnosed by either CT, US, or MRI; (2) the gallbladder showed satisfactory function by US (with the estimated gallbladder ejection fraction greater than 30 % or at stage I or II; and (3) no stone incarceration was found; (4) the patients were unwilling to receive LC or medically unfit for LC. Exclusion criteria were: (1) gallbladder atrophy and thickening of the cyst wall; (2) coagulopathy; (3) the stone in the cyst duct; and (4) other reasons that may cause the obstruction of the cystic duct. All patients provided informed consent for the procedure. This study was approved by the board of the Shengjing Hospital of China medical University (NO.2015PS16J).

### Devices

**Fig. 1:**
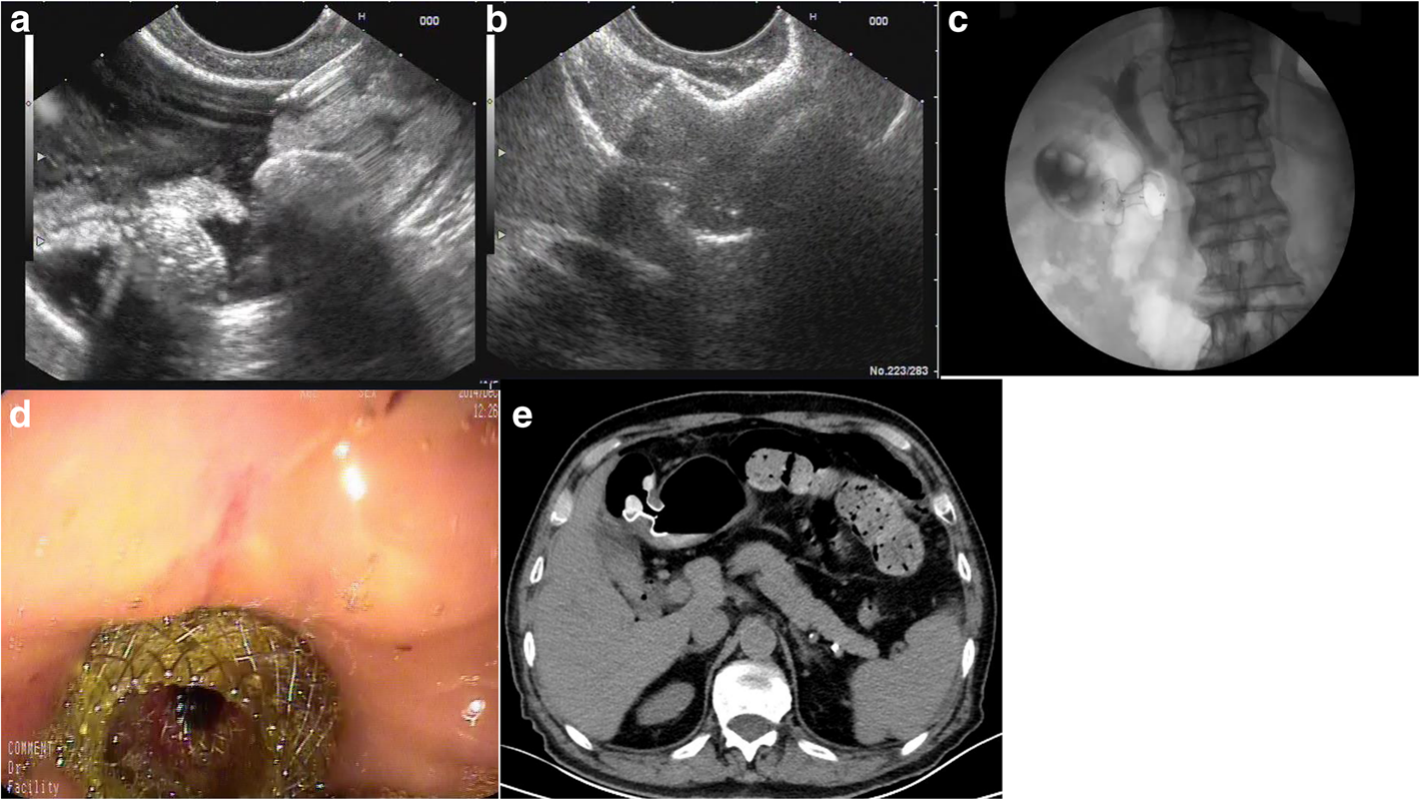
Cholecystogastrostomy. **a** EUS guided gallbladder puncture by echo-tip needle. **b** EUS guided stent releasing. **c** X-ray image of the released sent. **d** endoscopic view of the released stent. **e** CT exam flowing the cholecystogastrostomy

Longitudinal echoendoscope (PENTAX EG3870UT, Pentax Corporation, Japan) with a working channel of 3.8 mm accessible to a 10Fr stent was used. Echo-Tip needle (19-G, Wilson-Cook Medic, USA) with a lumen of 0.8 mm in diameter was fitted to a 0.035 in. guidewire (Tracer metro direct wire guide, 0.035 in. /480 mm, Wilson Cook Medical Inc, USA). Cystotome (10Fr, Wilson-Cook Medic) was used to dilate the tract and create a large fistula. The stent (Micro-Tech/Nan Jing CO.Ltd. China) had a self-expanding nitinol mesh design, with 2 large flared ends. Fully expanded, the 2 ends measured 20 mm in diameter. The waist of the stent measured 10 mm in diameter and the stent was 35 mm long. The 2 large flared ends were designed to protrude against the adjacent luminal walls with moderate pressure. The stent was fully covered by a polyester membrane, which prevents leakage and allows easily retrieval (Fig. 1). The OTSC (over the scope clips) device was produced by Ovesco Endoscopy AG, Tuebingen Germany. A nasobiliary drainage catheter (ENBD,7Fr, Wilson-Cook Medic) was used for gallbladder drainage.

### Procedure

#### EUS-guided Transmural cholecystoduodenostomy or cholecystogastrostomy (combined with EST therapy)

The EUS-guided cholecystogastrostomies and per-oral transgastric cholecystoscopic therapy were all performed by 1 skilled endoscopist. The patients was under general anesthesia. For the patients have both gallbladder stones and CBD stones, a standard EST procedure was performed first. After the CBD stones was cleared, a longitudinal echoendoscope with a working channel of 3.8 mm was introduced into the stomach or duodenal to scan for the gall bladder and to mark the puncture point. The contact zone (i.e., the region of the GI wall representing the shortest distance between the GI and gall bladder walls) was identified. Color Doppler was then used to identify interposing vessels in order to avoid these during the puncture. An EchoTip needle was introduced via the working channel of the echoendoscope, and the gallbladder was punctured under EUS guidance. A sample was aspirated to confirm that the punctured structure was gallbladder. 5 ml radiographic contrast (omnipaque) was injected into the gallbladder. Several loops of a guide wire were inserted into the gallbladder, and the needle was removed. The path of the needle was dilated using a Cystotome.

Under EUS and fluoroscope guidance, the stent was slowly deployed into the gallbladder and released until the flared distal end was completely open. Gentle traction was applied to pull the gallbladder wall close to the gastric wall. Then, under endoscopic surveillance, the remainder of the stent was deployed, keeping the proximal end in sight. EUS was used to confirm the position of the stent and to rule out leakage. After the stent placement, antibiotics (Ceftriaxone) were administered intravenously after the procedure for 48 h. The oral diet was suspended for 48 h. If no complications were observed 48 h after the procedure the patient’s normal diet could be resumed.

#### Per-oral transgastric cholecystoscopic therapy (cholecystolithotomy or cholecystic polyps resection)

One or two weeks after the cholecystogastrostomy with metal stent, the fistula between the GI tract and the gallbladder had formed. Either CT or X-ray was used to determine that the stent remained in place. The cholecystoscopy technique could then be performed with the metal stent in place or removed. A dilation balloon could be applied to dilate the tract, if the outer diameter of the endoscope used to insert into the gallbladder was greater than 8 mm. The endoscope was advanced into the gallbladder via the fistula formed by the stent. A stone basket was inserted into the gallbladder to remove the stones. For the patients had polyps, a snare or argon plasma coagulation (APC) can be used for the resection of polyps, and EMR technique could also be applied.

Following the procedure within the gallbladder, an ENBD (endoscopic nasobiliary drainage) is placed in the gallbladder, and 24 h later, radiography performed via the ENBD tube to check if there are any residual stones. EGD with transparent cap was applied to check the healing of the fistula 24 h after the ENBD is removed. If the GI end of of the fistula was closed, the patient’s normal diet could be resumed.

### Follow up

Four weeks after per-oral transgastric cholecystoscopy, the contractile response of the patient’s gallbladder should be assessed by abdominal ultrasound. The gallbladder functions are classified according to a fat meal test (Table 1). Regular US exam should be done at 1, 3, 6 and 12 months after the treatment in the first year and every 12 months in the following years.

**Table 1:** Gallbladder function classification (fat meal test)

Grade	Empty	30 min	60 min	90 min
I	Well-filling cyst of normal size and shape	obvious	>1/2	>3/4
II	Mild changes in the morphology of cyst	mild	>1/3	>1/3
III	Gallbladder shrinkage and wall thickening (4–5 mm)	no	<1/3	<1/3
IV	Atrophy of the cyst	no	no	no

## Results

A total of 10 patients with symptomatic gallbladder stone treated at Shengjing Hospital between June 2013 and February 2015 were enrolled in this study. 2 patient showed gallbladder function of level III was excluded from this study and the other patient revealed the stone in the cyst duct were also excluded. 7 patients (3 males and 4 females), with ages ranging from 60 to 86 years old, were selected for this procedure (Table 2). 5 patients were found to have gallbladder stones, 2 patients has both gallbladder stones and polyps. Five of these patients complicated by the presence of common bile duct stones. These diagnoses were all confirmed by CT,MRI or US.

**Table 2:** The characteristic of the patients

Mean age (Y)	73.2 (65–85)
Gender	
Male	3
Female	4
Gallstones	7
Polyps	2
CBD stones	5
Diameter of the stones	5–15 mm
Symptoms	
Abdominal pain	7
Jaundice	5
Fever	3

**Table 3:** Cholecystogastrostomy

^a^Mean time (mins)	9.8
Location	
Antrum	4
Duodenal bulb	3
Success rate	7/7
Increased leucocytes	7/7
Fever	4/7
Hemorrhage	0/7
Severe peritonitis	0/7
Displaced	0/7

^a^Time: The whole time for the cholecystogastrostomy procedure

**Table 4:** Per-oral transgastric cholecystoscopic therapy

Duration (days)	9.1
Formation of the fistula	7/7
Dilation of the fistula	2/7
Gallstone removed	7/7
Polyp resection	2/2
Stone residual	0/7
Delayed healing	1/7
Other complications	0/7

Cholecystogastrostomy was successfully performed in 2 patients and cholecystogastrostomy combined with EST therapy was also successfully performed in the other 5 patients who were complicated with common bile duct stones (Fig. 1) (Table 3). Most of the patients (6/7) complained of mild abdominal discomfort following this anastomosis procedure. One patient complained obvious abdominal pain and distention. The emerging CT scanning revealed the dilation of the stomach and water retention. The depressing tube was inserted into the stomach and release the pain. All 7 patients experienced transient increases in Blood leucocytes and 4 developed mild fever, which recovered within 24 h. No signs of peritonitis were observed, and the patients resumed a normal diet 24 h after the procedure.

**Fig. 2:**
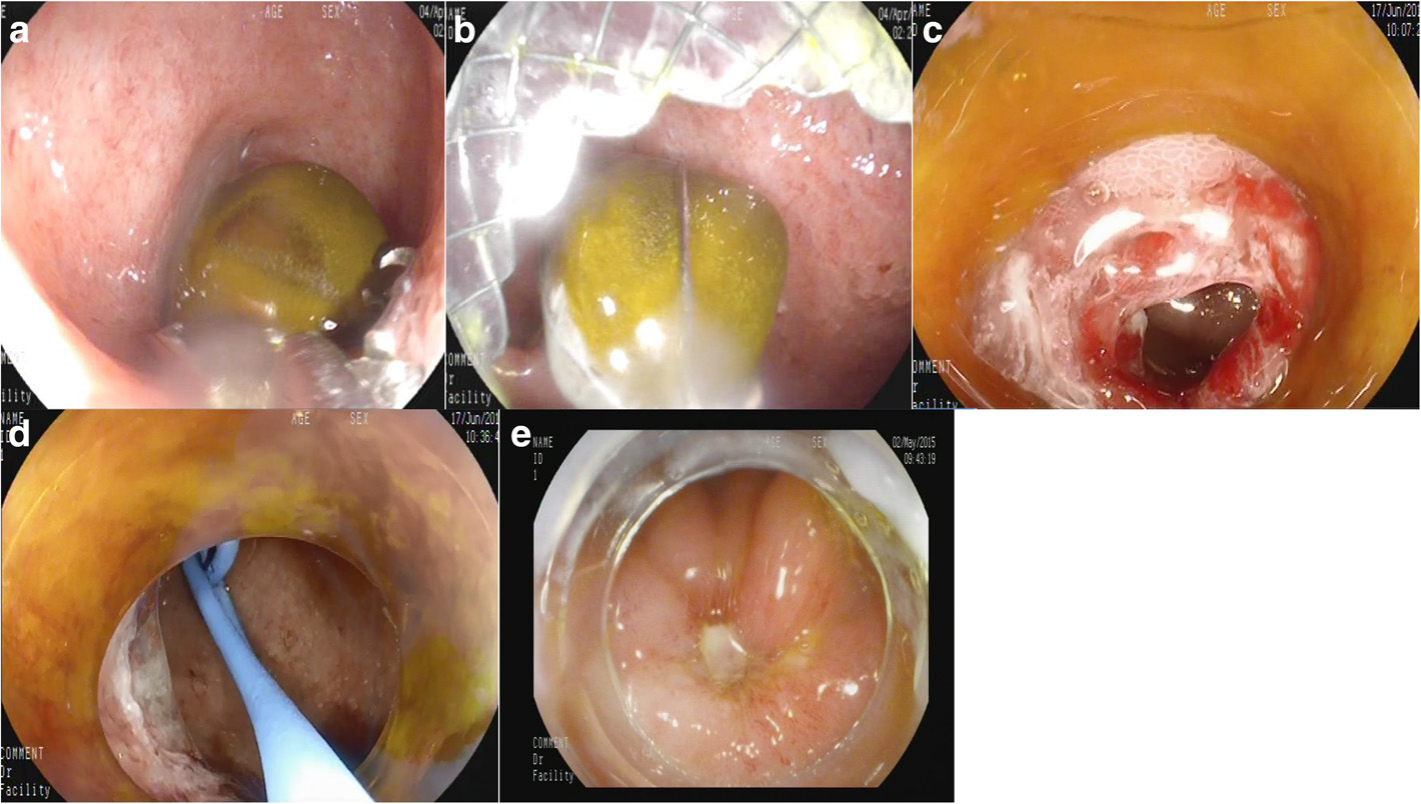
Gastric transmural cholecystolithotomy and polyps resection. **a** Stone was revealed in the gallbladder by the endoscope. **b** Stone was removed from the gallbladder. **c** Fistula formed by the metal stent (stent was removed). **d** ENBD was inserted into the gallbladder through the fistula. **e** The fistula was closed 3 days following the ENBD removing and ulcer was formed

Cholecystolithotomy was successfully performed in 7 patients (Table 4), and cholecystic polyp resection was successfully performed in 2 patients (Fig. 2). The metal stents were all removed by forceps in 7 patients; this was done after the transgastric gallbladder therapy in 5 patients and before the procedure begins in 2 patients. An ENBD tube was placed in the gallbladder. Twenty-four hours later, radiography performed via the ENBD tube showed that the gallbladder was free of residual stones for the 7 gallbladder stone patients. The pathology of the polyps from the 2 patients identified these as cholesterol polyps.

**Fig 3:**
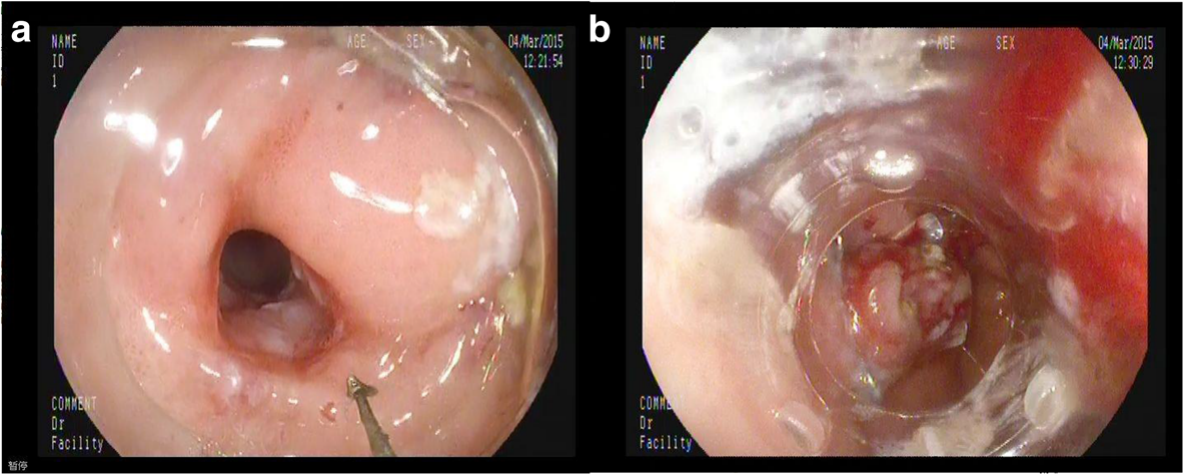
Fistula closing by OTSC. **a** Un-healing of the fistula. **b** The fistula was closed by OTSC

The fistula was completely self-closed in 6 patients within 24 h after the removal of the ENBD (checked by EGD). In one patient, the fistula remained unhealed. Three days after the cholelithotomy and stent removal, a large amount of food residual was deposited in the gallbladder with the fistula unclosed, as revealed by endoscopic examination. An OTSC device was successfully used to close the fistula after food residual in the gallbladder was cleaned (Fig. 3).

Four weeks after transgastric cholecystolithotomy, the contractile response of the patient’s gallbladder was assessed by abdominal ultrasonography. The gallbladder ejection fraction was estimated ranging from 80 to 91 % (86 % on average), which indicated satisfactory functioning at Level 1. During a following up of 3-20months, no stone recurrence was found in these patients.

## Discussion

Choledocholithiasis is a common problem that for almost the past 300 years has been treated by surgery [6]. A major step in the treatment of choledocholithiasis was the evolution from laparotomy to laparoscopy, which today remains the standard treatment for gallstones. Long-term follow-up studies have found that cholecystectomy is associated with an increased incidence of dyspepsia, calculus of the common bile duct, and colon carcinoma. There are also serious complications associated with the procedure, including hemorrhage, bile duct injury, bile leakage, and abdominal infection [11–13]. For these patients whose still have good gallbladder functions, gallbladder-preserving cholecystolithotomy should be considered. Laparoscopic, transcutaneous and endoscopic methods were reported in the previous studies [14–18]. In our study, EUS-guided trans-GI tract cholecystolithotomy has the advantages of sampler, preciser and less invasive (scarless).

Between 10 and 18 % of patients undergoing laparoscopic cholecystectomy (LC) for gallbladder stones also have concurrent common bile duct (CBD) stones [19]. The most popular approach to treating CBD stones detected prior to LC is with ERCP, followed by LC [20]. This two-stage approach has some disadvantages, including the risk of CBD stone passage taking place between ERCP and LC or during LC due to excessive gallbladder handling. These potential problems can be avoided by using the single-session laparoscopic approach for managing CBD stones during LC by transcystic exploration (TCE) or laparoscopic CBD exploration (LCBDE), which is as safe and effective as the ‘gold standard’ sequential ERCP followed by LC with the nearly same rate of success, length of hospital stay, and rate of complications [10]. In this study, we used a trans-gastrointestinal tract cholecystoscopy technique in treating GB disease without cholecystectomy, which may be superior in reduction of residual scarring and in preserving of gallbladder function. We also combined EST and cholecystolithotomy into a one-session procedure for those patients with both gallstones and common bile duct stones, which may become another popular alternative treatment option besides LC plus LCBDE.

Transgastric gallbladder entry through a cholecystogastric fistula formed as a complication following gallbladder surgery was first described in a case report by Chen et al. However, cholecystoenterostomy may also be a complication of gallstone disease rather than of medical intervention [21]. EUS has the ability to locate abdominal structure sprecisely [22]. In a previous animal study, we evaluated EUS-assisted transmural cholecystolithotomy performed 4 weeks after acholecystogastrostomy procedure in which a novel, fully covered metal stent was inserted in place, guided by EUS [23, 24]. Transmural cholecystogastrostomy using a lumen-apposing metal stent may not only be used to facilitate gallbladder drainage, but it may also be a technically easy method for treating cholelithiasis and polyps. The efficient anastomosis formation that we found in our study could be attributed to the lumen-apposing metal stent, which has 2 large, flared ends that firmly hold the gastric wall against the gallbladder wall. The blunted edges of the flared ends act to prevent further injury to the mucosa. The covered stents were also found to prevent bile leakage. Over the past 3 years reports of several types of lumen-apposing metal stents have been published; these have mainly found application in endoscopic pancreatic fluid collection drainage, gallbladder drainage and common bile duct drainage [25–31]. EUS-guided transmural cholecystolithotomy by a metal stent has been reported by Itoi et al., with the stone removal performed through a lumen-apposing metal stent in a patient with acute cholecystitis, rather than through a fistula maturely formed by stent or intended to be applied in patients with good gallbladder function [32]. In our case, we used this technique as a bridge to facilitate per-oral cholecystoscopic therapy.

In our clinical study, the EUS-guided cholecystogastrostomies were all successfully performed. 7 patients experienced transient increases in Blood leucocytes and 4 developed mild fever. These incidents may have been caused by limited leakage of bile taking place during the procedure,which however did not develop into peritonitis. Other severe complications such as pneumoperitoneum, hemorrhage and dislocation of the stent, were not observed in the patients in this study, which indicates the safety of this procedure.

The optimal timing of the cholecystoscopic technique following the cholecystogastrostomy depends on the maturity of the fistula formed by the stent. It is safe to allow for 7 days for the fistula dilation, removal of gallstones and polyp resection. The fistulas were expected to completely heal within 24 h after the ENBD was removed. However, there was 1case of delayed healing of the fistula found in our study; this fistula was located at the lesser curvature of the antrum near the pylorus. Since, in this case, food debris always filled the gallbladder, we assumed that the fistula in this location may have interfered with the gastric emptying function. A trans-duodenal path may have less impact than a transgastric path in the gastric emptying function, which could therefore be a better place of puncture. However, this assumption needs further evaluation. An OTSC device was used to help in closing the fistula. According to the literature, the use of OTSC in the closing of a fistula is not very common [33–35].

It is noteworthy that the ultrasound follow-up evaluation of 20 month showed no gallstone or polyps recurrence and the function of the gallbladder remained satisfactory. Hence this endoscopic gallbladder-preserving cholecystolithotomy or polyp resection may be an option for patients with gallstones or polyps but who still have a well-functioning gallbladder.

### Limitations

Reflux of gastric contents into the gallbladder through the stent may increase the risk of infection and stone formation. Therefore, the lumen-apposing metal stent should be further modified so that it has an anti-reflux function. In addition, it remains unknown as to whether the pylori function would be interfered with by the location of the fistula, so further studies are needed in this regard. Finally, studies with a larger sample size and a longer follow-up time are required.

## Conclusion

The EUS-guided placement of a novel metal stent is a safe and simple approach to performing an endoscopic cholecystogastrostomy, which can subsequently allow for procedures for treating biliary disease, including cholecystolithotomy and polyp resection. Although we cannot conclude from this case that EUS-guided cholecystolithotomy will replace laparoscopic cholecystectomy and become the procedure of choice for gallstones, it does have obvious advantages, especially for the patient with a gallbladder that is functioning well. It is a minimally-invasive, scarless procedure that preserves the gallbladder and its digestive functions.
